# The School Board Elections

**Published:** 1900-11-10

**Authors:** 


The Hospital

A Journal of

The Medical Sciences and Hospital Administration.

Vol. XXIX.—No. 737.] Edited by Sir Henry Burdett, K.C.B., and Solomon C. Smith, M.D., M.R.C.P. [November 10, 1900.

### The School Board Elections.

The frankly political character of the recent municipal elections in the twenty-eight boroughs into which the metropolis is now divided must, we think, be a source of considerable satisfaction to all by whom a prospect of improved local government is valued; and may probably be taken as an indication that the coming elections to the School Board will be conducted on similar lines. There has, we know, been a great deal of arguing that education is a non-political question; and the same thing was said about the functions of the County Council; but, in both cases, chiefly with a view that political partisans of the most declared character might put on sheep's clothing for the occasion, and might divest themselves of it as soon as the object for which it was assumed had been secured. The same party names will, of course, be used by different people, and on different occasions, to connote very different opinions; and therefore, as a rule, it is mere question begging to employ them. To a political opponent, either one party name or the other expresses only an archetype, the supposed copies of which may be of almost infinite variety, and may present the widest differences from the imaginary original; but still the names are used as if they indicated something, and we read and speak of Conservatives without asking what they desire to conserve, of Progressives without asking whither they would progress, of Radicals without asking to what root their attention is directed, and of Moderates without asking whether their moderation may not resemble that of Eli the High Priest, whose sons made themselves vile, and he restrained them not. As a matter of fact, the Conservative party of to-day holds very different views, on many subjects, from those which were held by the same party, so-called, even ten years ago. The School Board for London must, in many respects, furnish an example to the School Boards of smaller communities; and its election ought clearly now to be conducted on party lines. There can be no doubt but that many candidates will disclaim politics, as they have done aforetime; but surely the net is spread in vain in the sight of any bird. The "independent" candidate, whoever he may be, is a free lance; while the political candidate is pledged to his party as a supporter of their policy, and, together with the party as a whole, must stand or fall by it. The heads of the party organisation must be held responsible for the selection of the candidates whom they approve and are prepared to support; and the success or failure of the policy of the Board for the next three years must be regarded as the success or failure of the party which has been mainly instrumental in framing and conducting it. Success would demand a renewal of the confidence of the ratepayers; failure would justify its withdrawal, and, probably, its transference to the political opponents of those by whom it had been forfeited. As the country is now governed, there is no other way in which any coherent policy can be secured. The individuals constituting a fortuitous assemblage of atoms may be all that can be desired; but, without the check which party ties are calculated to furnish, they will almost of necessity be found pulling in a variety of different ways.

An important problem which the new Board will have to solve, in some practical fashion, will be the best means of providing for the education of feeble-minded and of crippled children; two classes which must be kept absolutely distinct, although they present the one point of resemblance of requiring special and usually separate treatment. With regard to both, important medical or physiological questions become prominent; and one of the chief defects of the present School Board is the absence of medical knowledge among its members. We believe there is not a single physician or surgeon on the Board; and the power of spending the education rate in the payment of salaried medical officers is extremely restricted. It is manifest, nevertheless, that a special school for either cripples or the feeble-minded would require constant medical supervision, as a condition essential to the attainment of the best possible results. In both cases, the very possibility of "overstrain" should be excluded. In the case of the feeble-minded, of course, all that can as a rule be hoped for is to rescue the children from their disabilities in a sufficient degree to enable them to earn, or to contribute towards, their own maintenance without becoming nuisances to those around them; but, in the case of the cripples, there is no limit to the amount of capacity which, in Huxley's admirable phrase, may be "caught" by judicious schooling, and so turned to account for the benefit alike of its possessors and of the public. We referred last week to the admirable work in this direction which has been done by Mrs. Humphry Ward at her "Settlement," and which she described in a paper read before the recent Conference of Women Workers. The results of her experiment have been sufficiently good, and sufficiently decisive, to justify the School Board in taking extensive action upon similar lines; and we trust that the next three years may see something in this direction fairly initiated and set in working order. There may be great differences of opinion with regard to the value of much of the teaching of the continuation schools to many of those who receive it; but there can, we should imagine, be none with regard to the advisability of assisting, by such teaching as they can receive and assimilate, those who are handicapped in the struggle for existence by infirmities which have been produced by accident or disease.

